# An unusual osteoma in the mandibular condyle and the successful replacement of the temporomandibular joint with a custom-made prosthesis: a case report

**DOI:** 10.1186/s13104-017-3060-4

**Published:** 2017-12-08

**Authors:** Natalia Tavares de Souza, Renan Carlos Lopes Cavalcante, Maria Aparecida de Albuquerque Cavalcante, Wagner Hespanhol, Marcello Rodrigues de Oliveira, Dennis de Carvalho Ferreira, Thais Machado de Carvalho Coutinho, Lucio Souza Gonçalves

**Affiliations:** 1 Federal University of Rio de Janeiro, Rio de Janeiro, RJ Brazil; 2 Faculty of Dentistry, Veiga de Almeida University, Rio de Janeiro, Brazil; 3 Faculty of Dentistry, Estácio de Sá University, Av. Alfredo Baltazar da Silveira, 580/cobertura, Recreio, Rio de Janeiro, RJ 22790-710 Brazil

**Keywords:** Osteoma, Benign tumor, Total temporomandibular joint, Prosthesis

## Abstract

**Background:**

An osteoma is a benign tumor of bone with unknown etiology and is considered rare, mostly restricted to the craniofacial skeleton.

**Case presentation:**

This case report describes an uncommon condylar osteoma in a 67 years old white female patient with laterognathism to the left side, limited mouth opening, aesthetic change and pain associated with the right temporomandibular joint (TMJ). The histopathological examination confirmed osteoma. The lesion was surgically excised and immediate reconstruction was carried out using a custom-made total TMJ prosthesis. The patient has been in follow-up for 2 years, with no symptoms.

**Conclusions:**

Unilateral total TMJ prosthesis can be considered to replacement of TMJ after osteoma excision with resection of the condyle.

## Background

An osteoma is defined as a benign tumor of bone resulting from the continuous formation of cortical and spongious bone. The etiology of the tumor is unknown, but may be associated with trauma, response to infections or inflammatory processes and growth abnormalities. Osteomas are rare and mostly restricted to the craniofacial skeleton. When they appear in the jaw region, there is a preference for the mandible rather than the maxilla. The most affected areas of the mandible are the body, angle and condyle [1–3].

Osteomas in the mandible have been reported as a cause of trismus, limitation of mouth opening, progressive malocclusion with midline shift, contralateral mandibular deviation and facial asymmetry, especially when the mandibular condyle is involved. They may be symptomatic when their growth surpasses the limits of the bone [2–4]. Research shows that men are affected two times more than women (2:1), with ages ranging from 14 to 58 years, with an average age of 29.4 years. Large osteomas that cause symptoms or esthetic deformities are excised surgically. Reconstruction using autogenous bone grafts or prosthetic joints is usually performed when the region of the mandibular condyle is affected [1–4]. The indications for total replacement of the temporomandibular joint (TMJ) are more than two previous TMJ surgeries, fibrous or bony ankylosis of the TMJ, and postoperative condylar loss associated to neoplastic excision [5–7].

A total TMJ prosthesis must be designed so that it minimizes biomechanical stresses and at the same time achieves a homeostatic equilibrium [8–10]. Finite-element modeling (FEM) has also been used to analyze the stress distribution in TMJ components [11, 12].

The custom-made total TMJ prosthesis “Promm” which is made in Brazil and is registered with ANVISA No. 10447390006 has been designed considering functional rehabilitation, anatomy and aesthetics of the patient.

This study reports a case of an uncommon condylar osteoma that was identified due to an aesthetic change of the TMJ and immediate reconstruction using a total TMJ prosthesis.

## Case presentation

**Fig. 1:**
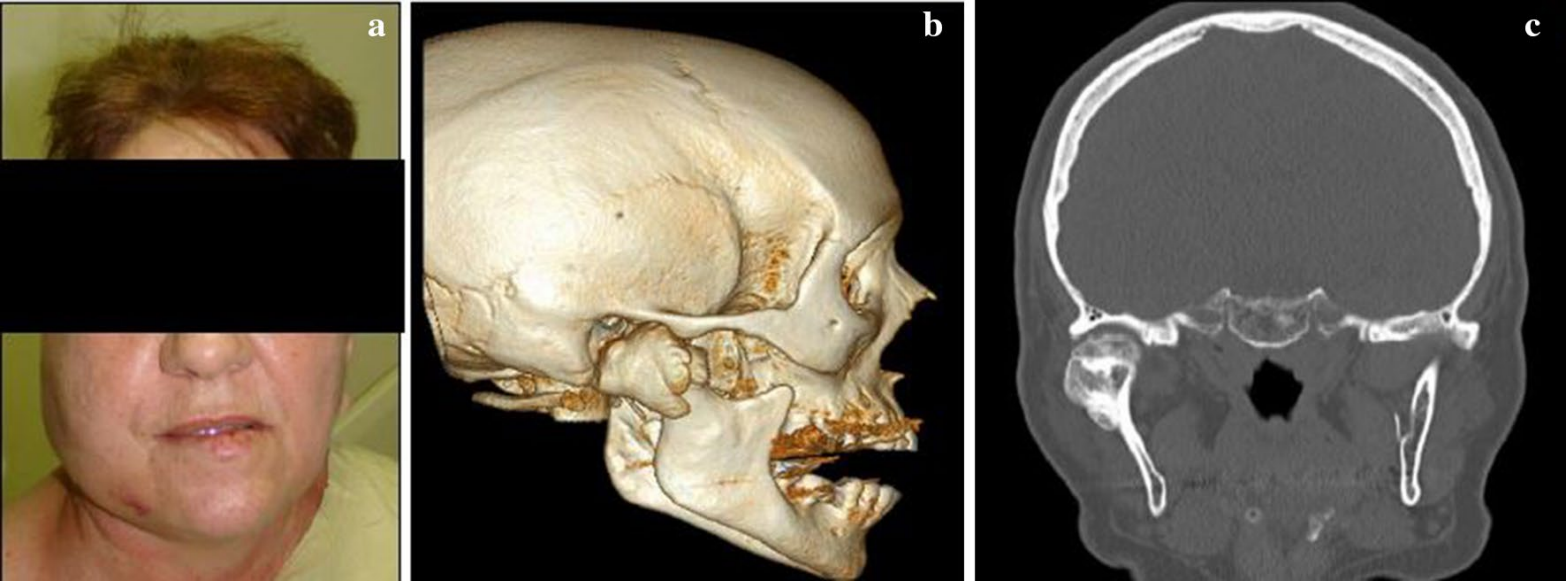
**a** The asymmetry in the left lower jaw of the patient; **b**, **c** 3D CT, note the large tumor on the right condyle, from two different angles

A 67-year-old white female patient was referred for the evaluation of asymmetry in the left lower jaw. On examination, the patient had laterognathism to the left side, limited mouth opening and pain in the right TMJ during maximum mouth opening and palpation. She reported that she had sought medical help due to extensive headaches. The doctor referred her to a dentist specialized in oral and maxillofacial surgery. The patient also reported the facial deviation that was becoming more and more noticeable to other people, interfering with her aesthetics (Fig. 1a). The patient made it clear that she had had no experience of previous trauma in the TMJ region.

Following the initial examination, a 3D CT scan was performed and it revealed an extensive but well-defined radiopaque lesion on the right condyle, measuring 1.5 by 2.0 cm (Fig. 1b, c). Thus, due to the size of the lesion, the decision was to excise the tumor with resection of the condyle and to construct a custom-made total temporomandibular joint prosthesis for reconstruction. The engineer and the surgeons responsible for the surgery discussed the project of the prosthesis and the availability of soft tissue, and the need for symmetry and aesthetics were assessed. This type of prosthesis consist of a component representing the glenoid fossa, which was made of ultra-high molecular weight polyethylene cast with pure titanium and is fixed to the mandibular fossa with titanium bolts. The component representing the condyle was made of a molybdenum cobalt-chromium alloy and the titanium branch [13, 14].

**Fig. 2:**
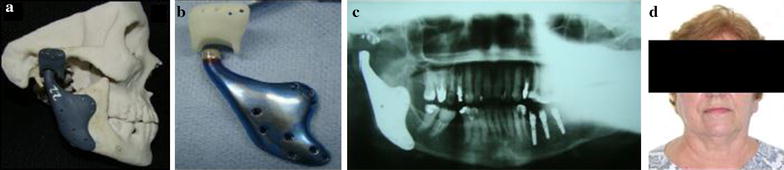
**a** Prototyping with the adapted TMJ prostheses; **b** TMJ prostheses image; **c** panoramic X-ray 2-year control; **d** patient image 2 years after surgery

The accurate prototype of the patient’s skull allowed a three-dimensional analysis of the lesion and was of significant assistance throughout the process. A CT scan was used to prepare the prototype and the custom-made prosthesis for the patient (Fig. 2a, b). For surgery, the patient underwent general anesthesia with endotracheal intubation.

Initially the movements of the mandible were studied using Posselt’s diagram. This device tracks the components of the cranial cavity of the prosthesis. The model of the prosthesis in resin was approved by the surgeon and scanned three dimensionally (3D). A computer numerical control (CNC) program used these images to machine the final model of the prosthesis. The mandibular component of the Arthroplasty System Promm consists of a condylar head made of cobalt–chromium–molybdenum alloy (ISO 5832-12) and a mandibular body made of pure titanium (ISO 5832-2), while the cranium component has a cavity where the condyle is articulated and machined in polyethylene of ultra-high molecular weight, UHMWPE (ISO 5834-2).

All parts received a finishing process. The mandibular component was submitted to electro coloring, resulting in a blue color. All Promm products are batch mark, which allows the traceability of the raw materials the batch, provider, trader and date of each step, as well as the tools used for quality control of the parts.

After performing asepsis, antisepsis and installation of drapes, the incisions were marked with a Skin Marker. At the location of the incisions lidocaine 2% with 1:50,000 epinephrine was infiltrated to improve homeostasis during the incision. The prosthetic joint was placed via the preauricular access and the submandibular incision access was used to fix the branch ramus and condyle. After exposure of the entire mandibular ramus, osteotomy was made with an incision safety margin until the trailing edge of the mandibular ramus, through the submandibular access, so that the condyle with the tumor could be removed via the preauricular access.

**Fig. 3:**
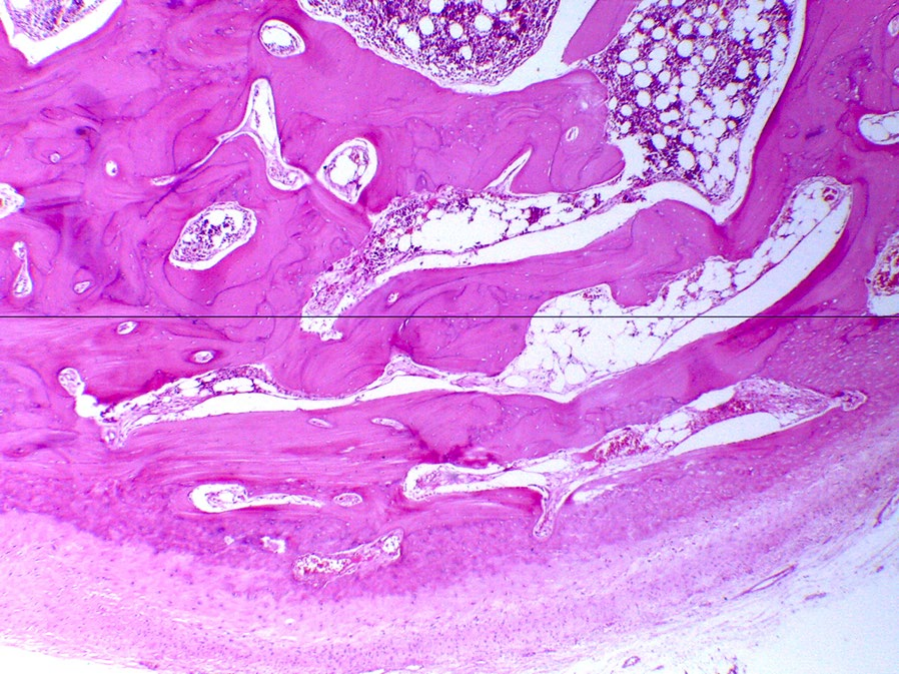
Compact bone tissue with fibrous connective stroma at the periphery (hematoxylin–eosin; original magnification, ×40)

**Fig. 4:**
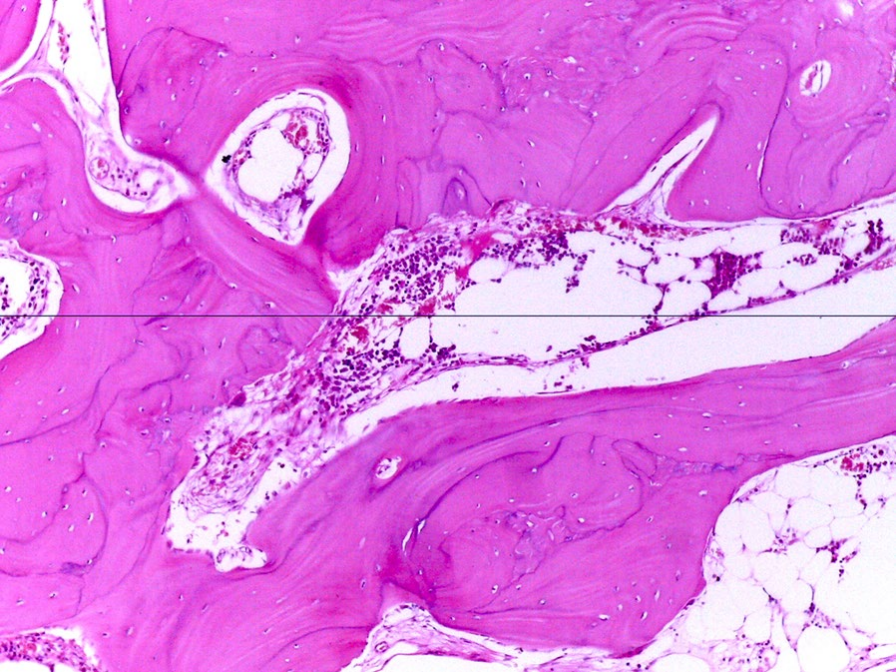
Compact bone with lacuna filled by bone marrow tissue (hematoxylin–eosin; original magnification, ×100)

The incised specimen was sent for histopathological examination, which confirmed osteoma. Figure 3 depicts a compact bone tissue with fibrous connective stroma at the periphery, while Fig. 4 presents compact bone with lacuna filled by bone marrow tissue.

After removal of the tumor, the prosthesis was adapted without difficulty and fixed in place with the 2.4 mm screw system. After implantation of the prosthesis the opening and closing mouth movements were tested and were found to be within normal limits. The patient has been in follow-up for 2 years, with no painful symptoms, mouth opening of 32 mm and she continues as an outpatient (Fig. 2c, d).

## Discussion and conclusions

The etiology of osteoma is unknown. Many authors have suggested that the majority of those in the maxillofacial region were reactive bone hyperplasia or advanced ossification. Other authors consider trauma as an important factor. However, in this case report these possibilities were not considered to be the etiologic factors [1–4, 15]. Osteomas, in most cases are asymptomatic, depending on their location and size. Lesions tend to be small, solitary, slow-growing and painless, and are only noticeable during routine examinations. As these lesions have a progressive characteristic, they eventually becoming larger and exacerbate the signs and symptoms. They can cause facial asymmetry, contralateral mandibular deviation, limitation of mouth opening and be painful [16, 17]. These manifestations are similar to those observed in the patient of this report.

Normally osteomas appear in isolation and alone. However, there is a syndrome which is associated with the appearance of multiple osteomas, called Gardner syndrome [3]. This syndrome is an autosomal dominant disorder, which has: multiple osteomas (especially in the facial bones and long bones), epidermoid cysts on the skin, connective tissue tumors, colorectal polyps with a great propensity of malignant transformation, supernumerary teeth as dental changes and malignant thyroid neoplasm [2, 3, 16]. According to the site where the tumors are formed, can be considered central or peripheral. In this case report, the patient did not show these signs and symptoms described in the literature.

As a differential diagnosis of these lesions, maxillary or mandibular exostoses, osteoid osteoma, osteoblastoma, chondroma, cemento-ossifying fibroma (COF), and odontoma may be mentioned. Palatal and mandibular torus are exostoses which cannot be regarded as osteomas, although they are histologically identical [4, 15].

Radiographically, osteomas show a well-circumscribed image with an oval or round radiopaque mass. Despite a radiographic image as described above, conclusive diagnosis can only be confirmed with a histopathologic examination [1, 4].

Treatment for osteomas consists in complete surgical removal of the base containing the cortical bone. Recurrence is rare, but periodic clinical follow-ups are recommended as well as radiographic exams after surgical excision [3]. Our patient has been in follow up for 2 years postoperatively without any complaints concerning mouth opening, pain or asymmetries.

The first case of a condylar osteoma was described by Ivy in 1927 [18]. Nowadays, large osteomas that cause symptoms or esthetic deformities are excised surgically. When the region of the mandibular condyle is affected, these should be reconstructed using autogenous bone grafts or prosthetic joints.

Reconstruction of TMJ is a complex surgical procedure and it entails improved mandibular form and function, reduction of pain and disability, containment of excessive treatment and cost as well as and the prevention of further morbidity [13]. The selection of patients presents a great challenge, since they all have different needs. Total TMJ replacement using alloplastic prosthesis may provide satisfactory results in cases of functional alterations of the TMJ due to the presence of tumors and diseases such as advanced forms of arthritis, ankylosis, and developmental anomalies with irreversible joint damage [6, 7, 19]. Aagaard and Thygesen [20] highlights the benefits of using custom made TMJ prosthesis based on orthopedic and biomechanical principles as a safe and efficient option when the patient presents a wide range of temporomandibular disorders. Park et al. [7] also demonstrated suitable outcomes of four patients who used custom made TMJ prostheses. Other on the hand, a TMJ prosthesis is expensive and success depends on the technique and implant used, especially if the option is the customized model.

In conclusion, successful replacement of a TMJ with a custom-made total temporomandibular joint prosthesis after surgical removal of a benign tumor is possible. Unilateral total TMJ prosthesis can be considered as a replacement of a TMJ after osteoma excision with resection of the condyle.

## Abbreviations

TMJ: temporomandibular joint
COF: cemento-ossifying fibroma
CNC: computer numerical control
